# The diagnostic performance of AFP and PIVKA-II models for non-B non-C hepatocellular carcinoma

**DOI:** 10.1186/s13104-023-06600-y

**Published:** 2023-11-06

**Authors:** Vinh Thanh Tran, Thang Thanh Phan, Tran Bao Nguyen, Thao Thi Le, Thanh-Tram Thi Tran, Anh-Thu Thi Nguyen, Hang Thuy Nguyen, Ngoc-Diep Bui Nguyen, Toan Trong Ho, Suong Phuoc Pho, Thuy-An Thi Nguyen, Hue Thi Nguyen, Huyen Thi Mai, Bich-Tuyen Thi Pham, Khoa Dinh Nguyen, Binh Thanh Le, Thuc Tri Nguyen, Son Truong Nguyen

**Affiliations:** 1 The Laboratory D Unit, Cancer Center, Cho Ray Hospital, #201B Nguyen Chi Thanh Street, Dist. 5, Ho Chi Minh City, 700000 Vietnam; 2 Department of Clinical Pathology, Cho Ray Hospital, #201B Nguyen Chi Thanh Street, Dist. 5, Ho Chi Minh City, 700000 Vietnam; 3 Scientific Research Department, Cho Ray Hospital, #201B Nguyen Chi Thanh Street, Dist. 5, Ho Chi Minh City, 700000 Vietnam; 4 Department of General Director, Cho Ray Hospital, #201B Nguyen Chi Thanh Street, Dist. 5, Ho Chi Minh City, 700000 Vietnam

**Keywords:** AFP, AFP-L3, PIVKA-II, NBNC-HCC, Diagnosis

## Abstract

**Objective:**

This study aims to describe the diagnostic performance of alpha-fetoprotein (AFP), alpha-fetoprotein L3 isoform (AFP-L3), protein induced by vitamin K absence II (PIVKA-II), and combined biomarkers for non-B non-C hepatocellular carcinoma (NBNC-HCC).

**Results:**

A total of 681 newly-diagnosed primary liver disease subjects (385 non-HCC, 296 HCC) who tested negativity for the hepatitis B surface antigen (HBsAg) and hepatitis C antibody (anti-HCV) enrolled in this study. At the cut-off point of 3.8 ng/mL, AFP helps to discriminate HCC from non-HCC with an area under the curve (AUC) value of 0.817 (95% confidence interval [CI]: 0.785–0.849). These values of AFP-L3 (cut-off 0.9%) and PIVKA-II (cut-off 57.7 mAU/mL) were 0.758 (95%CI: 0.725–0.791) and 0.866 (95%CI: 0.836–0.896), respectively. The Bayesian Model Averaging (BMA) statistic identified the optimal model, including patients’ age, aspartate aminotransferase, AFP, and PIVKA-II combination, which helps to classify HCC with better performance (AUC = 0.896, 95%CI: 0.872–0.920, *P* < 0.001). The sensitivity and specificity of the optimal model reached 81.1% (95%CI: 76.1–85.4) and 83.2% (95%CI: 78.9–86.9), respectively. Further analyses indicated that AFP and PIVKA-II markers and combined models have good-to-excellent performance detecting curative resected HCC, separating HCC from chronic hepatitis, dysplastic, and hyperplasia nodules.

**Supplementary Information:**

The online version contains supplementary material available at 10.1186/s13104-023-06600-y.

## Introduction

HCC is the third leading cause of cancer-related death worldwide, which is related most to viral hepatitis B and C infections (80–90%) [1, 2]. Other factors associated with HCC development are alcohol consumption, aflatoxin B1, non-alcoholic fatty liver disease (NAFLD), non-alcoholic steatohepatitis (NASH), type 2 diabetes, obesity, and smoking [1, 3]. For the treatment benefit in the early stage, repeated screening of HCC is recommended in all at-risk patients, especially those with hepatitis virus infections and liver cirrhosis [3]. The pathological assessment is the current gold-standard method that requests tumors from biopsy or surgical procedures. Besides, diagnostic imaging tools such as ultrasonography, computed tomography, and magnetic resonance are used widely in HCC surveillance. Limitations of these techniques are that equipment quality and operators’ experience can affect diagnostic results.

Some biomarkers are used frequently in HCC screening, including HBsAg, hepatitis B core antibody, anti-HCV, albumin, bilirubin, prothrombin, complete blood count, AFP, and PIVKA-II [3–5]. Numerous studies have shown that AFP and PIVKA-II serum proteins are accurate biomarkers for HCC surveillance [4–6]. However, this referred information is for the mixed etiology or viral hepatitis. A few studies assessed the diagnostic role of these markers in those without hepatitis B and C viruses (non-B non-C HCC, NBNC-HCC) [7–12], which tends to rise in current years [13, 14]. We aim to clarify the role of AFP, AFP-L3, PIVKA-II, and other markers in the surveillance of NBNC-HCC by real-world data.

## Materials and methods

### Study cohort and biomarker measurement

**Fig. 1:**
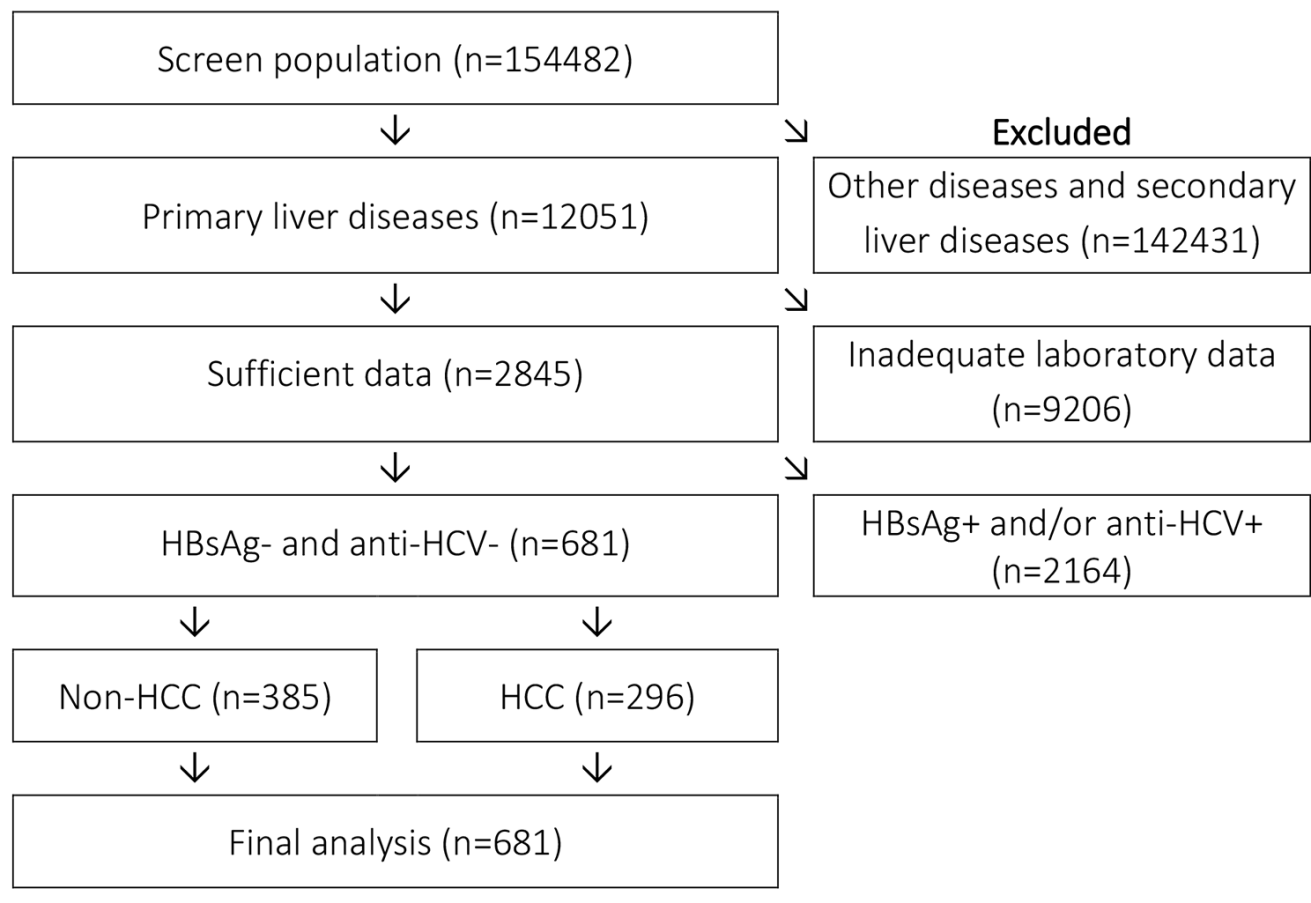
Patient selection

In total, 681 consecutively newly diagnosed patients (from May 2015 to Dec-2022) enrolled in this study (Fig. 1). The inclusion criteria were: primary liver diseases confirmed by histopathological assessment; HBsAg- and anti-HCV-; and availability of laboratory data within one week before performing biopsy or surgery, including AFP, AFP-L3, PIVKA-II, aspartate aminotransferase (AST), alanine aminotransferase (ALT), albumin, bilirubin, glycemia, blood nitrogen urea (BUN), creatinine, activated partial thromboplastin time (APTT), fibrinogen, prothrombin time (PT), and cell blood count parameters. Among 681 subjects, 385 cases were classified as non-HCC, including chronic hepatitis, liver cirrhosis, simple hepatic cyst, hyperplasia nodule, low-high grade dysplastic nodule, intrahepatic stones, necrosis, and normality results. Two hundred and ninety-six remaining cases were confirmed with HCC. Details of the study cohort were presented in the Additional file 1: Table S1. This study was performed in accordance with the Declaration of Helsinki and was reviewed and approved by the Ethics Committees of Cho Ray Hospital (approval number: 1496-GCN-HDDD). The written informed consent was waived due to the retrospective study.

The AFP, AFP-L3, and PIVKA-II concentrations were measured by the µTASWako platform (FUJIFILM Wako GmbH, Neuss, Germany) or the ARCHITECT i2000SR (Abbott Laboratories, Illinois, USA). The biochemical tests, blood cell count, and coagulation parameters were done with the ARCHITECT c16000, Alinity-HQ (Abbott Laboratories, Illinois, USA), and STA R Max (Stago, Inc., NJ, USA), respectively.

### Statistical analysis

We used the Wilcoxon rank-sum test to compare the median value of each marker between groups. The receiver operating characteristic curve was constructed and defined the optimal cut-off point, then calculated diagnostic values (AUC value, sensitivity, and specificity) of each marker in screening HCC. Moreover, the BMA statistic was used to select biomarkers and build an optimal model for HCC diagnosis. Besides, the accuracy of the model was appraised by the calibration test. All data analyses were done with R statistical software v.4.2.2 (R foundation, 1020 Vienna, Austria). *P* < 0.05 was considered statistically significant.

## Results

### Patients’ characteristics

**Fig. 2:**
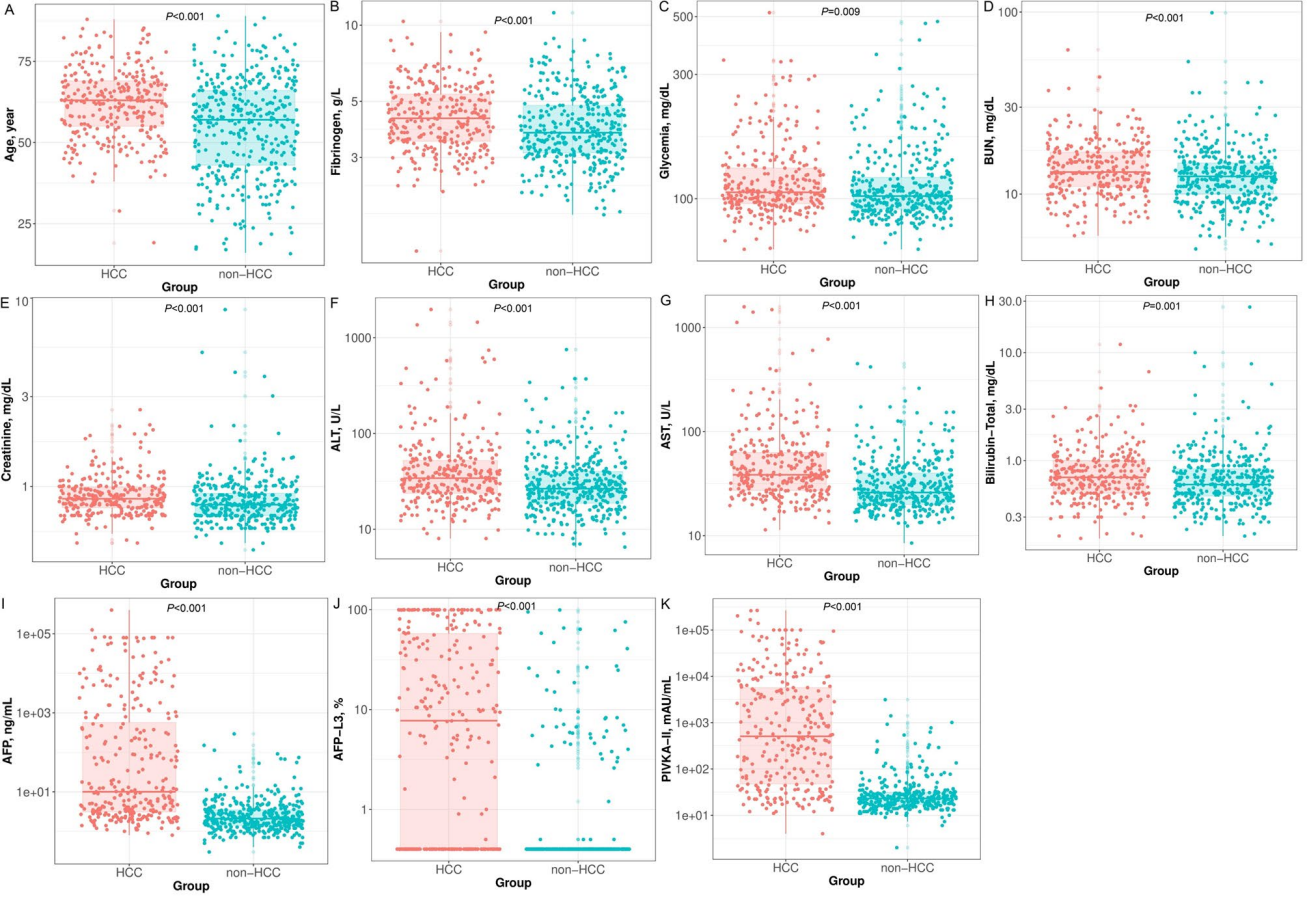
Distribution of age, fibrinogen, glycemia, BUN, creatinine, ALT, AST, bilirubin, AFP, AFP-L3, and PIVKA-II expression between non-HCC and HCC patients (**A**-**K**)

All 681 patients (235 female and 446 male) enrolled in this study were negative with HBsAg and anti-HCV markers. The median age of non-HCC was 57 years which was lower than HCC (63 years, *P* < 0.001). The diagnostic tests at presentation showed that fibrinogen, glycemia, BUN, creatinine, ALT, AST, and bilirubin concentrations were significantly higher in HCC compared to non-HCC (Additional file 2: Table S2 and Fig. 2). Also, HCC patients have a higher level of AFP, AFP-L3, and PIVKA-II concentration (*P* < 0.001).

### The diagnostic performance of markers for HCC

**Fig. 3:**
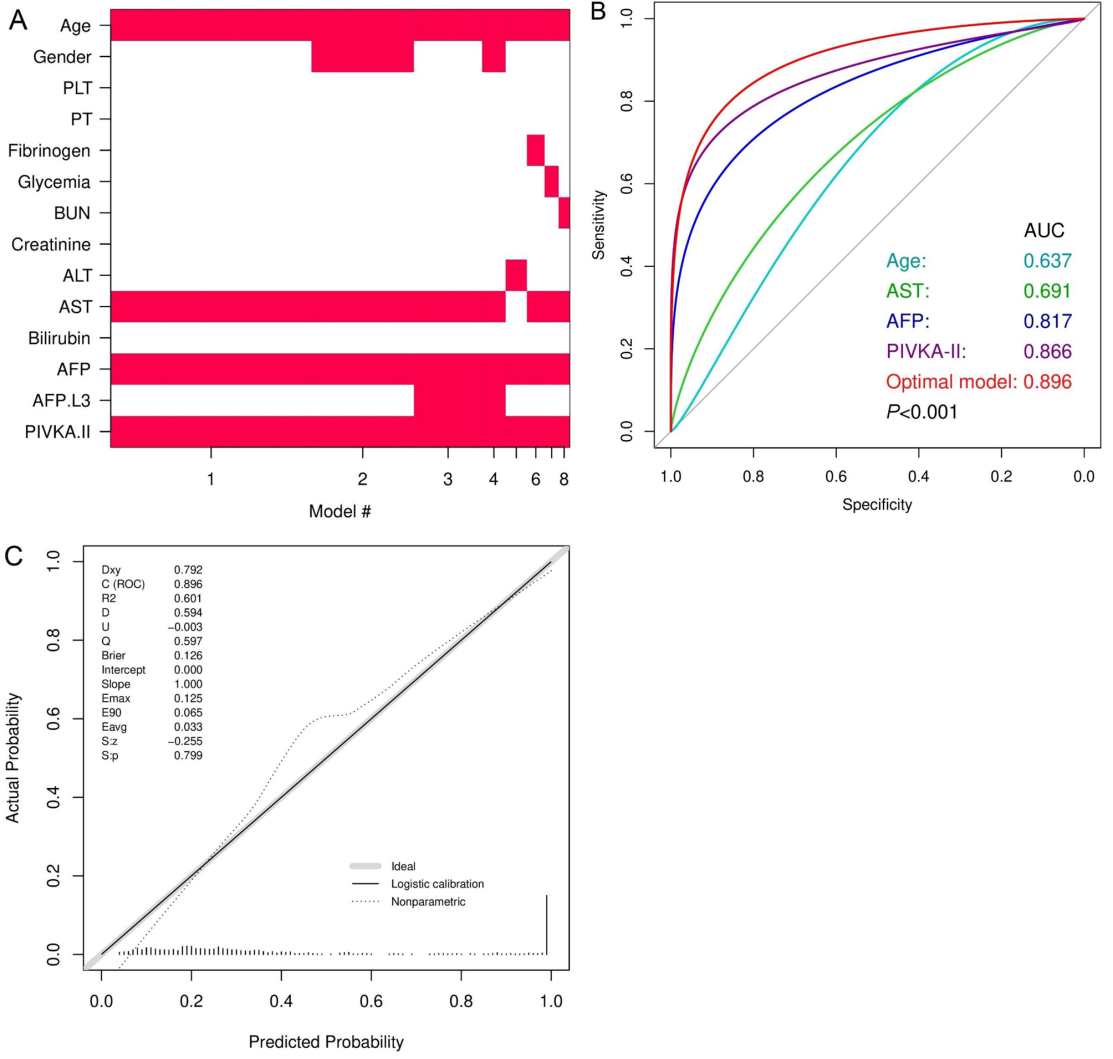
Selection of diagnostic tests by the BMA statistic (**A**), the performance of optimal model versus individual markers (**B**), and calibration analysis (**C**)

The diagnostic values of markers for NBNC-HCC are shown in Additional file 3: Table S3. The analyses demonstrated that a high level of AFP serum protein (≥ 3.8 ng/mL) helps to distinguish HCC from primary liver diseases with the AUC value of 0.817 (95%CI: 0.785–0.849), 68.6% sensitivity (95%CI: 62.9–73.8), and 78.2% specificity (95%CI: 73.7–82.2). These values of AFP-L3 (cut-off 0.9%) were 0.758 (95%CI: 0.725–0.791), 61.0% (95%CI: 55.1–66.6), and 87.0% (95%CI: 83.2–90.2), respectively. For the PIVKA-II performance (cut-off 57.7 mAU/mL), an AUC value of 0.866 (95%CI: 0.836–0.896), 73.9% sensitivity (95%CI: 68.4–78.8), and 88.8% specificity (95%CI: 85.1–91.9) were observed. Moreover, the combination of AFP and PIVKA-II improved diagnostic performance (AUC = 0.887, 95%CI: 0.860–0.914) compared to the single biomarker (*P* < 0.001). Notably, We found the highest AUC value (0.896, *P* < 0.001) resulted from the AFP + PIVKA-II + AST + Age combined model that was identified by the multivariable logistic regression (Fig. 3A and B). The sensitivity and specificity of this model reached 81.1% (95%CI: 76.1–85.4) and 83.2% (95%CI: 78.9–86.9), respectively. Positive and negative values are acceptable (79.7% and 84.4%, Additional file 3: Table S3). Calibration analysis with a small Brier score (0.126) indicated that this optimal model is accurate in clinical practice (Fig. 3C).

In subgroup analyses, AFP, PIVKA-II, AFP + PIVKA-II, and AFP + AFP-L3 + PIVKA-II + ALT + Age models showed good capabilities in differentiating HCC from chronic hepatitis (AUC values were 0.814, 0.869, 0.885, and 0.922, respectively, Additional file 4: Table S4). Similarly, in separating HCC from dysplastic and hyperplasia nodules, AFP and PIVKA-II models served with AUC values above 0.81 (Additional file 5: Table S5). Furthermore, We investigated marker capacity for those undergoing tumor resection and noted an extremely high performance of PIVKA-II (AUC = 0.930), AFP + PIVKA-II (AUC = 0.936), and AFP + PIVKA-II + AST models (AUC = 0.966, *P* = 0.044, Additional file 6: Table S6).

## Discussion

NBNC-HCC is a small proportion of total HCC but has been increasing continuously [13, 14]. Without awareness of HBsAg and anti-HCV markers, HCC screening in communities becomes difficult. It is more arduous when most cases do not have any signs or symptoms before detecting HCC at the advanced stages [14]. AFP, AFP-L3, and PIVKA-II are important biomarkers (low-cost, non-invasiveness, rapid and easy implementation, standardization, and accepted performance) that have been globally recognized and applied for many years, but few studies dealt with the NBNC-HCC group [7–12]. We investigated and showed that the AFP + PIVKA-II model combined with AST and patients’ age exhibited good diagnostic accuracy, sensitivity, specificity, and positive and negative predictive values in classifying NBNC-HCC (Additional file 3: Table S3). These results are comparable to the data of mixed etiologies and viral HCC detection [4–6]. This model is different from the findings of Nouso K (FIB4A model: AST + ALT + platelets + age + AFP) [7], Best J (GALAD model: age + gender + AFP + AFP-L3 + PIVKA-II) [9], Caviglia GP (age + gender + PIVKA-II + glypican-3 + adiponectin) [10], and Guan MC (AFP + PIVKA-II) [11]. It is easy to see that subjects of these studies (diabetes, NAFLD, and NASH) are different from our populations (chronic hepatitis, simple hepatic cyst, necrosis, hyperplasia, dysplastic nodules…), which may lead to distinct observations. Importantly, analyses indicated that our model is accurate clinically based on good classification capacity, sensitivity, specificity, small Brier score, and fitted actual values with predicted probabilities (Fig. 3C). These results suggest clinicians should request AFP and PIVKA-II parallel to routine tests (AST, ALT, albumin, bilirubin…) and ultrasound in regular examinations. This integration can allow us to detect NBNC-HCC earlier, thus improving prognosis and long-term survival for patients.

Impressively, We found that AFP and PIVKA-II models separate NBNC-HCC well from hyperplasia and dysplastic nodules, which are pre-cancerous stages, progress to the HCC in several months to years [15, 16]. To our knowledge, this has not been shown in any studies before. Additionally, AFP and PIVKA-II models display excellent performance in distinguishing resected tumor HCC (Additional file 6: Table S6), comparable to the observation of Li Y [8].

## Conclusion

The results of this study indicated that patients’ age and fibrinogen, glycemia, BUN, creatinine, ALT, AST, bilirubin, AFP, AFP-L3, and PIVKA-II serum levels are higher in NBNC-HCC compared to the non-HCC. Besides, the AFP + PIVKA-II model combined with patients’ age and AST can help classify NBNC-HCC with good performance.

### Limitations

This study highlights the diagnostic power of AFP and PIVKA-II models for NBNC-HCC by analysis of a large cohort, however, have some limitations. Because of a retrospective study, We did not collect enough data from medical records such as imaging results, clinical stage, and metastasis variable, which help categorize patients and further analyses. In addition, treatment therapy with vitamin K, which affects PIVKA-II serum concentration, was not collected. A prospective study with adequate clinical variables should be conducted to confirm our findings.

### Electronic supplementary material

Below is the link to the electronic supplementary material.

Supplementary Material 1

Supplementary Material 2

Supplementary Material 3

Supplementary Material 4

Supplementary Material 5

Supplementary Material 6

## Data Availability

All data generated or analysed during this study are included in this published article (Additional file 1: Table S1).

## Abbreviations

95%CI: 95% confidence interval
AFP: Alpha-fetoprotein
AFP-L3: Alpha-fetoprotein L3 isoform
ALT: Alanine aminotransferase
anti-HCV: Hepatitis C antibody
APTT: Activated partial thromboplastin time
AST: Aspartate aminotransferase
AUC: Area under the curve
BMA: Bayesian model averaging
BUN: Blood nitrogen urea
HBsAg: Hepatitis B surface antigen
HGB: Hemoglobin
MCV: Mean corpuscular volume
NAFLD: Non-alcoholic fatty liver disease
NASH: Non-alcoholic steatohepatitis
NBNC-HCC: Non-B non-C hepatocellular carcinoma
NPV: Negative predictive value
PIVKA-II: Protein induced by vitamin K absence II
PLT: Platelets
PPV: Positive predictive value
PT: Prothrombin time
RBC: Red blood cells
WBC: White blood cells

## References

[1] Sung H, Ferlay J, Siegel RL, Laversanne M, Soerjomataram I, Jemal A, Bray F (2021). Global cancer statistics 2020: GLOBOCAN estimates of incidence and mortality worldwide for 36 cancers in 185 countries. CA Cancer J Clin.

[2] El-Serag HB (2012). Epidemiology of viral hepatitis and hepatocellular carcinoma. Gastroenterology.

[3] Vogel A, Cervantes A, Chau I, Daniele B, Llovet JM, Meyer T (2018). Hepatocellular carcinoma: ESMO clinical practice guidelines for diagnosis, treatment and follow-up. Ann Oncol.

[4] Zhang J, Chen G, Zhang P, Zhang J, Li X, Gan D (2020). The threshold of alpha-fetoprotein (AFP) for the diagnosis of hepatocellular carcinoma: a systematic review and meta-analysis. PLoS ONE.

[5] Fan J, Chen Y, Zhang D, Yao J, Zhao Z, Jiang Y (2020). Evaluation of the diagnostic accuracy of des-gamma-carboxy prothrombin and alpha-fetoprotein alone or in combination for hepatocellular carcinoma: a systematic review and meta-analysis. Surg Oncol.

[6] Pang BY, Leng Y, Wang X, Wang YQ, Jiang LH (2023). A meta-analysis and of clinical values of 11 blood biomarkers, such as AFP, DCP, and GP73 for diagnosis of hepatocellular carcinoma. Ann Med.

[7] Nouso K, Furubayashi Y, Shiota S, Miyake N, Oonishi A, Wakuta A (2020). Early detection of hepatocellular carcinoma in patients with Diabetes Mellitus. Eur J Gastroenterol Hepatol.

[8] Li Y, Chen Y, Chen J (2020). Diagnostic value of serum biomarkers for patients undergoing curative resection with non-B, non-C hepatocellular carcinoma. J Coll Physicians Surg Pak.

[9] Best J, Bechmann LP, Sowa JP, Sydor S, Dechêne A, Pflanz K (2020). GALAD score detects early hepatocellular carcinoma in an international cohort of patients with nonalcoholic steatohepatitis. Clin Gastroebterol Hepatol.

[10] Caviglia GP, Armandi A, Rosso C, Gaia S, Aneli S, Rolle E (2021). Biomarkers of oncogenesis, adipose tissue dysfunction and systemic inflammation for the detection of hepatocellular carcinoma in patients with nonalcoholic fatty Liver Disease. Cancers (Basel).

[11] Guan MC, Ouyang W, Liu SY, Sun LY, Chen WY, Tong XM (2022). Alpha-fetoprotein, protein induced by vitamin K absence or antagonist-II, lens culinaris agglutinin-reactive fraction of alpha-fetoprotein alone and in combination for early detection of hepatocellular carcinoma from nonalcoholic fatty Liver Disease: a multicenter analysis. Hepatobiliary Pancreat Dis Int.

[12] Liu D, Luo Y, Chen L, Chen L, Zou D (2021). Diagnostic value of 5 serum biomarkers for hepatocellular carcinoma with different epidemiological backgrounds: a large-scale, retrospective study. Cancer Biol Med.

[13] Younossi ZM, Stepanova M, Ong J, Trimble G, AlQahtani S, Younossi I (2021). Nonalcoholic steatohepatitis is the most rapidly increasing indication for liver transplantation in the United States. Clin Gastroenterol Hepatol.

[14] Tateishi R, Uchino K, Fujiwara N, Takehara T, Okanoue T, Seike M (2019). A nationwide survey on non-B, non-C hepatocellular carcinoma in Japan: 2011–2015 update. J Gastroenterol.

[15] Kudo M (2009). Multistep human hepatocarcinogenesis: correlation of imaging with pathology. J Gastroenterol.

[16] Ng CH, Chan SW, Lee WK, Lai L, Lok KH, Li KK (2011). Hepatocarcinogenesis of regenerative and dysplastic nodules in Chinese patients. Hong Kong Med J.

