# Supplementary material for: The diagnostic performance of AFP and PIVKA-II models for non-B non-C hepatocellular carcinoma
Source: BMC Res Notes. 2023 Nov 6;16:317. doi: 10.1186/s13104-023-06600-y (PMC10629103; doi:10.1186/s13104-023-06600-y)
Supplement: Supplementary file 2 — Supplementary Material 2 [file 13104_2023_6600_MOESM2_ESM.docx]

**Table S2.** Characteristics of included patients

| **Variable** | **Total (n=681)** | **Non-HCC (n=385)** | **HCC (n=296)** | ***P*-value** |
| --- | --- | --- | --- | --- |
| Median age, year (95%CI) | 60 (58-61) | 57 (55-59) | 63 (61-64) | *<0.001* |
| Gender: female/male | 235/446 | 167/218 | 68/228 | *<0.001* |
| RBC, 10^12^/L (95%CI) | 4.47 (4.42-4.53) | 4.50 (4.43-4.55) | 4.44 (4.34-4.59) | 0.592 |
| HGB, g/L (95%CI) | 132 (131-134) | 131 (129-134) | 133 (131-135) | 0.087 |
| MCV, fL (95%CI) | 90.9 (90.3-91.6) | 90.6 (89.9-91.4) | 91.5 (90.6-92.5) | 0.062 |
| WBC, 10^9^/L (95%CI) | 8.2 (7.9-8.5) | 8.0 (7.7-8.4) | 8.4 (8.0-8.8) | 0.237 |
| PLT, 10^9^/L (95%CI) | 253 (246-260) | 260 (250-273) | 244 (231-255) | *0.013* |
| PT, s (95%CI) | 13.1 (13.1-13.2) | 13.1 (13.0-13.1) | 13.3 (13.1-13.4) | *0.025* |
| APTT, s (95%CI) | 29.3 (29.1-29.6) | 29.3 (29.1-29.6) | 29.4 (28.9-29.7) | 0.516 |
| Fibrinogen, g/L (95%CI) | 3.96 (3.85-4.08) | 3.76 (3.61-3.92) | 4.28 (4.06-4.49) | *<0.001* |
| Glycemia, mg/dL (95%CI) | 104 (102-106) | 102 (101-105) | 106 (103-108) | *0.009* |
| BUN, mg/dL (95%CI) | 13.0 (12.5-13.1) | 12.5 (12.0-13.0) | 13.2 (13.0-14.1) | *<0.001* |
| Creatinine, mg/dL (95%CI) | 0.82 (0.80-0.84) | 0.80 (0.79-0.81) | 0.86 (0.84-0.88) | *<0.001* |
| ALT, U/L (95%CI) | 29.0 (27.4-30.7) | 26.5 (25.0-28.0) | 34.1 (31.0-37.1) | *<0.001* |
| AST, U/L (95%CI) | 31.0 (29.3-33.0) | 26.0 (24.6-28.0) | 38.5 (35.9-42.0) | *<0.001* |
| Albumin, g/dL (95%CI) | 4.13 (4.08-4.18) | 4.15 (4.10-4.20) | 4.08 (4.01-4.18) | 0.073 |
| Bilirubin-T, mg/dL (95%CI) | 0.66 (0.62-0.70) | 0.60 (0.59-0.66) | 0.70 (0.67-0.75) | *0.001* |
| AFP, ng/mL (95%CI) | 3.1 (2.8-3.4) | 2.1 (1.9-2.2) | 10.0 (6.5-15.9) | *<0.001* |
| AFP-L3, % (95%CI) | 0.4 (0.3-0.4) | 0.4 (0.3-0.4) | 7.7 (4.7-10.8) | *<0.001* |
| PIVKA-II, mAU/mL (95%CI) | 32.0 (29.4-36.2) | 23.0 (22.0-24.5) | 506.0 (328.9-860.8) | *<0.001* |

**Abbreviations:** 95%CI, 95% confidence interval; AFP, Alpha-fetoprotein; AFP-L3, Alpha-fetoprotein L3 isoform; ALT, Alanine aminotransferase; AST, Aspartate aminotransferase; APTT, Activated partial thromboplastin time; BUN, Blood nitrogen urea; HGB, Hemoglobin; MCV, Mean corpuscular volume; PIVKA-II, Protein induced by vitamin K absence II; PLT, Platelets; PT, Prothrombin time; RBC, Red blood cells; WBC, White blood cells.

**Notes:** data were presented as median (95%CI).
