# Supplementary material for: The diagnostic performance of AFP and PIVKA-II models for non-B non-C hepatocellular carcinoma
Source: BMC Res Notes. 2023 Nov 6;16:317. doi: 10.1186/s13104-023-06600-y (PMC10629103; doi:10.1186/s13104-023-06600-y)
Supplement: Supplementary file 3 — Supplementary Material 3 [file 13104_2023_6600_MOESM3_ESM.docx]

**Table S3.** Performance of individual markers in diagnosis NBNC HCC

| **Variable** | **Cut-off** | **AUC (95%CI)** | **Sensitivity, % (95%CI)** | **Specificity, % (95%CI)** | **PPV, % (95%CI)** | **NPV, % (95%CI)** |
| --- | --- | --- | --- | --- | --- | --- |
| Age, year | ≥44 | 0.637 (0.596-0.678) | 97.0 (94.3-98.6) | 26.8 (22.4-31.5) | 50.4 (46.2-54.6) | 91.9 (85.3-96.3) |
| ALT, U/L | ≥32.3 | 0.606 (0.563-0.649) | 53.7 (47.8-59.5) | 65.2 (60.2-69.9) | 54.3 (48.4-60.1) | 64.7 (59.7-69.5) |
| AST, U/L | ≥33.7 | 0.691 (0.652-0.731) | 62.5 (56.7-68.0) | 68.3 (63.4-72.9) | 60.3 (54.5-65.8) | 70.3 (65.4-74.9) |
| AFP, ng/mL | ≥3.8 | 0.817 (0.785-0.849) | 68.6 (62.9-73.8) | 78.2 (73.7-82.2) | 70.7 (65.1-75.9) | 76.4 (71.9-80.5) |
| AFP-L3, % | ≥0.9 | 0.758 (0.725-0.791) | 61.0 (55.1-66.6) | 87.0 (83.2-90.2) | 78.1 (72.1-83.3) | 74.5 (70.3-78.5) |
| PIVKA-II, mAU/mL | ≥57.7 | 0.866 (0.836-0.896) | 73.9 (68.4-78.8) | 88.8 (85.1-91.9) | 84.3 (79.3-88.5) | 80.7 (76.4-84.4) |
| AFP+PIVKA-II^†^ | ≥-0.876 | 0.887 (0.860-0.914) | 73.5 (68.1-78.5) | 93.3 (90.2-95.6) | 89.9 (85.4-93.4) | 81.2 (77.1-84.9) |
| Optimal model^‡^ | ≥-0.757 | 0.896 (0.872-0.920) | 81.1 (76.1-85.4) | 83.2 (78.9-86.9) | 79.7 (74.7-84.2) | 84.4 (80.2-88.0) |

**Abbreviations:** 95%CI, 95% confidence interval; AFP, Alpha-fetoprotein; AFP-L3, Alpha-fetoprotein L3 isoform; ALT, Alanine aminotransferase; AST, Aspartate aminotransferase; AUC, Area under curve; PIVKA-II, Protein induced by vitamin K absence II; PPV, Positive predictive value; NPV, Negative predictive value.

**Notes:** ^†^Y = -1.3380 + (0.01118*AFP) + (0.00301*PIVKA-II); ^‡^Y = -4.0210 + (0.04029*Age) + (0.00696*AST) + (0.00996*AFP) + (0.00279*PIVKA-II); only biomarkers with AUC≥0.6 have been shown.
