# Supplementary material for: The diagnostic performance of AFP and PIVKA-II models for non-B non-C hepatocellular carcinoma
Source: BMC Res Notes. 2023 Nov 6;16:317. doi: 10.1186/s13104-023-06600-y (PMC10629103; doi:10.1186/s13104-023-06600-y)
Supplement: Supplementary file 4 — Supplementary Material 4 [file 13104_2023_6600_MOESM4_ESM.docx]

**Table S4.** Performance of biomarkers in differentiating HCC from chronic hepatitis

| **Variable** | **Cut-off** | **AUC (95%CI)** | **Sensitivity, % (95%CI)** | **Specificity, % (95%CI)** | **PPV, % (95%CI)** | **NPV, % (95%CI)** |
| --- | --- | --- | --- | --- | --- | --- |
| Age, year | ≥44 | 0.664 (0.608-0.719) | 97.0 (94.3-98.6) | 28.9 (21.7-36.8) | 73.0 (68.3-77.4) | 82.7 (69.7-91.8) |
| BUN, mg/dL | ≥14.8 | 0.604 (0.549-0.658) | 41.9 (36.2-47.7) | 79.9 (72.5-86.0) | 80.5 (73.4-86.4) | 40.9 (35.2-46.8) |
| Creatinine, mg/dL | ≥0.81 | 0.609 (0.553-0.666) | 63.2 (57.4-68.7) | 55.7 (47.4-63.8) | 73.9 (68.0-79.2) | 43.2 (36.1-50.5) |
| ALT, U/L | ≥26.4 | 0.616 (0.562-0.671) | 66.9 (61.2-72.2) | 53.0 (44.7-61.2) | 73.9 (68.2-79.0) | 44.6 (37.2-52.3) |
| AST, U/L | ≥31.9 | 0.718 (0.667-0.768) | 66.2 (60.5-71.6) | 68.5 (60.4-75.8) | 80.7 (75.1-85.4) | 50.5 (43.4-57.6) |
| AFP, ng/mL | ≥3.2 | 0.814 (0.775-0.853) | 74.7 (69.3-79.5) | 73.2 (65.3-80.1) | 84.7 (79.7-88.8) | 59.2 (51.8-66.4) |
| AFP-L3, % | ≥0.9 | 0.770 (0.735-0.805) | 61.0 (55.1-66.6) | 87.8 (81.5-92.6) | 90.8 (85.9-94.5) | 53.3 (46.8-59.7) |
| PIVKA-II, mAU/mL | ≥80.0 | 0.869 (0.836-0.902) | 70.1 (64.5-75.3) | 93.8 (88.2-97.3) | 96.2 (92.7-98.4) | 58.2 (51.1-65.0) |
| AFP+PIVKA-II^†^ | ≥0.0336 | 0.885 (0.854-0.915) | 76.3 (71.0-81.1) | 92.2 (86.2-96.2) | 95.7 (92.2-97.9) | 63.3 (56.0-70.2) |
| Optimal model^‡^ | ≥0.4888 | 0.922 (0.897-0.946) | 80.1 (75.0-84.5) | 92.2 (86.2-96.2) | 95.9 (92.5-98.0) | 67.2 (59.8-74.1) |

**Abbreviations:** 95%CI, 95% confidence interval; AFP, Alpha-fetoprotein; AFP-L3, Alpha-fetoprotein L3 isoform; ALT, Alanine aminotransferase; AST, Aspartate aminotransferase; AUC, Area under curve; BUN, Blood nitrogen urea; PIVKA-II, Protein induced by vitamin K absence II; PPV, Positive predictive value; NPV, Negative predictive value.

**Notes:** ^†^Y = -0.6303 + (0.00771*AFP) + (0.00805*PIVKA-II); ^‡^Y = -4.1790 + (0.05112*Age) + (0.00925*ALT) + (0.00430*AFP) + (0.06041*AFP-L3) + (0.00680*PIVKA-II); only biomarkers with AUC≥0.6 have been shown.
