# Supplementary material for: The diagnostic performance of AFP and PIVKA-II models for non-B non-C hepatocellular carcinoma
Source: BMC Res Notes. 2023 Nov 6;16:317. doi: 10.1186/s13104-023-06600-y (PMC10629103; doi:10.1186/s13104-023-06600-y)
Supplement: Supplementary file 5 — Supplementary Material 5 [file 13104_2023_6600_MOESM5_ESM.docx]

**Table S5.** Performance of biomarkers in differentiating HCC from dysplastic and hyperplasia nodules

| **Variable** | **Cut-off** | **AUC (95%CI)** | **Sensitivity, % (95%CI)** | **Specificity, % (95%CI)** | **PPV, % (95%CI)** | **NPV, % (95%CI)** |
| --- | --- | --- | --- | --- | --- | --- |
| Age, year | ≥47 | 0.604 (0.548-0.659) | 93.2 (89.8-95.8) | 29.6 (23.0-36.9) | 68.7 (63.9-73.2) | 72.6 (60.9-82.4) |
| ALT, U/L | ≥34.1 | 0.601 (0.548-0.653) | 50.3 (44.5-56.2) | 67.0 (59.6-73.9) | 71.6 (65.0-77.6) | 44.9 (38.9-51.1) |
| AST, U/L | ≥33.7 | 0.668 (0.618-0.718) | 62.5 (56.7-68.0) | 65.9 (58.5-72.8) | 75.2 (69.3-80.5) | 51.5 (44.8-58.2) |
| AFP, ng/mL | ≥8.5 | 0.811 (0.774-0.848) | 52.7 (46.8-58.5) | 94.4 (90.0-97.3) | 94.0 (89.2-97.1) | 54.7 (49.0-60.3) |
| AFP-L3, % | ≥0.9 | 0.752 (0.715-0.788) | 61.0 (55.1-66.6) | 86.6 (80.7-91.2) | 88.1 (82.8-92.2) | 57.6 (51.5-63.6) |
| PIVKA-II, mAU/mL | ≥57.7 | 0.863 (0.831-0.896) | 73.9 (68.4-78.8) | 88.7 (83.1-93.0) | 91.5 (87.2-94.7) | 67.4 (60.9-73.4) |
| AFP+PIVKA-II^†^ | ≥-0.1246 | 0.885 (0.856-0.914) | 73.5 (68.1-78.5) | 93.8 (89.2-96.9) | 95.1 (91.4-97.5) | 68.3 (62.1-74.1) |
| Optimal model^‡^ | ≥0.1628 | 0.895 (0.868-0.922) | 75.6 (70.3-80.4) | 92.1 (87.1-95.6) | 94.0 (90.2-96.7) | 69.7 (63.3-75.5) |

**Abbreviations:** 95%CI, 95% confidence interval; AFP, Alpha-fetoprotein; AFP-L3, Alpha-fetoprotein L3 isoform; ALT, Alanine aminotransferase; AST, Aspartate aminotransferase; AUC, Area under curve; PIVKA-II, Protein induced by vitamin K absence II; PPV, Positive predictive value; NPV, Negative predictive value.

**Notes:** ^†^Y = -0.7816 + (0.02022*AFP) + (0.00382*PIVKA-II); ^‡^Y = -3.2410 + (0.03600*Age) + (0.00696*AST) + (0.01923*AFP) + (0.00359*PIVKA-II); only biomarkers with AUC≥0.6 have been shown.
