# Supplementary material for: The diagnostic performance of AFP and PIVKA-II models for non-B non-C hepatocellular carcinoma
Source: BMC Res Notes. 2023 Nov 6;16:317. doi: 10.1186/s13104-023-06600-y (PMC10629103; doi:10.1186/s13104-023-06600-y)
Supplement: Supplementary file 6 — Supplementary Material 6 [file 13104_2023_6600_MOESM6_ESM.docx]

**Table S6.** Performance of biomarkers for the curative resected HCC

| **Variable** | **Cut-off** | **AUC (95%CI)** | **Sensitivity, % (95%CI)** | **Specificity, % (95%CI)** | **PPV, % (95%CI)** | **NPV, % (95%CI)** |
| --- | --- | --- | --- | --- | --- | --- |
| Fibrinogen, g/L | ≥3.44 | 0.610 (0.553-0.667) | 83.3 (74.7-89.9) | 37.7 (32.8-42.7) | 26.1 (21.5-31.3) | 89.5 (83.7-93.8) |
| ALT, U/L | ≥32.7 | 0.696 (0.639-0.752) | 64.7 (54.6-73.9) | 65.5 (60.5-70.2) | 33.2 (26.7-40.2) | 87.5 (83.1-91.1) |
| AST, U/L | ≥33.8 | 0.763 (0.716-0.811) | 73.5 (63.9-81.8) | 68.6 (63.7-73.2) | 38.3 (31.4-45.5) | 90.7 (86.8-93.8) |
| AFP, ng/mL | ≥5.1 | 0.851 (0.808-0.893) | 69.6 (59.7-78.3) | 85.7 (81.8-89.1) | 56.3 (47.2-65.2) | 91.4 (88.0-94.1) |
| AFP-L3, % | ≥0.9 | 0.768 (0.717-0.820) | 64.4 (54.2-73.6) | 87.0 (83.2-90.2) | 56.5 (47.0-65.7) | 90.3 (86.8-93.1) |
| PIVKA-II, mAU/mL | ≥118.0 | 0.930 (0.895-0.965) | 84.8 (76.2-91.3) | 94.7 (91.8-96.8) | 81.6 (72.7-88.5) | 95.8 (93.1-97.6) |
| AFP+PIVKA-II^†^ | ≥-2.3276 | 0.936 (0.903-0.969) | 87.9 (79.8-93.6) | 93.6 (90.5-95.9) | 79.1 (70.3-86.3) | 96.5 (94.0-98.2) |
| Optimal model^‡^ | ≥-2.0603 | 0.966 (0.948-0.984) | 87.9 (79.8-93.6) | 93.3 (90.2-95.7) | 78.4 (69.6-85.6) | 96.5 (94.0-98.2) |

**Abbreviations:** 95%CI, 95% confidence interval; AFP, Alpha-fetoprotein; AFP-L3, Alpha-fetoprotein L3 isoform; ALT, Alanine aminotransferase; AST, Aspartate aminotransferase; AUC, Area under curve; PIVKA-II, Protein induced by vitamin K absence II; PPV, Positive predictive value; NPV, Negative predictive value.

**Notes:** ^†^Y = -2.7765 + (0.01266*AFP) + (0.00271*PIVKA-II); ^‡^Y = -3.3290 + (0.00984*AST) + (0.01095*AFP) + (0.00273*PIVKA-II); only biomarkers with AUC≥0.6 have been shown.
